# New Enhanced Artificial Bee Colony (JA-ABC5) Algorithm with Application for Reactive Power Optimization

**DOI:** 10.1155/2015/396189

**Published:** 2015-03-23

**Authors:** Noorazliza Sulaiman, Junita Mohamad-Saleh, Abdul Ghani Abro

**Affiliations:** ^1^School of Electrical & Electronic Engineering, Universiti Sains Malaysia, 14300 Nibong Tebal, Penang, Malaysia; ^2^College of Engineering, King Saud University, Muzahmyiah Campus, Riyadh 11451, Saudi Arabia

## Abstract

The standard artificial bee colony (ABC) algorithm involves exploration and exploitation processes which need to be balanced for enhanced performance. This paper proposes a new modified ABC algorithm named JA-ABC5 to enhance convergence speed and improve the ability to reach the global optimum by balancing exploration and exploitation processes. New stages have been proposed at the earlier stages of the algorithm to increase the exploitation process. Besides that, modified mutation equations have also been introduced in the employed and onlooker-bees phases to balance the two processes. The performance of JA-ABC5 has been analyzed on 27 commonly used benchmark functions and tested to optimize the reactive power optimization problem. The performance results have clearly shown that the newly proposed algorithm has outperformed other compared algorithms in terms of convergence speed and global optimum achievement.

## 1. Introduction

Bioinspired algorithms (BIAs) are metaheuristics method that imitates the biological phenomenon of nature [1, 2]. Various BIAs have been developed to solve complex optimization problems. For example, Davidović et al.  (2011) have implemented Bee Colony Optimization (BCO) algorithm to solve *p*-center problem [3] and, in 2012, Badar et al. have used particle swarm optimization (PSO) algorithm to handle a reactive power control problem [4]. Karaboga and Latifoglu then applied artificial bee colony (ABC) algorithm as a tool to solve adaptive filtering noisy transcranial Doppler signal [5]. Bacanin and Tuba (2014) have recently employed firefly algorithm to encounter cardinality constrained mean-variance portfolio optimization problem [6]. A few new BIAs have also been developed such as in the work of Obagbuwa and Adewumi that introduced Improved Cockroach Swarm Optimization (CSO) algorithm. The algorithm includes the insertion of hunger element to the existing CSO to enhance the exploration capabilities and the diversity of cockroach population [7]. Meanwhile, Zhou et al. (2014) have proposed Cloud Model Bat algorithm which is based on the ideas of bat echolocation together with the attribute of cloud model in order to depict good performance in optimization [8].

BIAs consist of several classes such as evolutionary algorithms (EA), swarm-intelligence-based (SI) algorithms, and many more. Among them, SI is the most prominent BIAs. SI algorithms imitate the social behavior of nature, such as bird flocking, fish schooling, and bees' swarming. SI has basically been a technique which is based on the interaction of organisms in a population, such as the flocks of bird and a swarm of bees. The optimization algorithms have been developed by observing the interaction among the swarm members [7]. Various optimization algorithms which are based on this technique have been successfully used in various optimization applications such as in real power loss minimization [4], estimation of induction motor's parameter [9], multilevel image thresholding [10], and many more. Among the techniques, optimization algorithms based on honeybees' behaviors have become the most commonly investigated and explored phenomenon by optimization researchers. Abbass (2001) has investigated marriage in honeybees [11]. Later on, Karaboga (2005) has proposed the artificial bee colony (ABC) algorithm based on the foraging behavior of honeybees [12]. Next, the concept of honeybees mating has been studied by Marinakis et al. [13] and Niknam et al. [14] in 2011. Besides that, the idea of the waggle dances of honeybees has been investigated by Duangphakdee et al. in 2011 who found out that the honeybees have complexity in waggle dances as soon as the sun comes close to its zenith. Thus, they have studied the relation of foraging and absconding to the azimuth [15].

ABC was proposed by Karaboga in 2005 [12]. It mimics the intelligent foraging behavior of honeybees that shows how organized the honeybees interact among them to search for food. ABC has fewer tuned parameters compared to other optimization algorithms such as genetic algorithm (GA) and differential evolution (DE). Thus, it is a simple and efficient optimization algorithm [16]. Moreover, ABC has been proven to show superior performance in comparison to other prominent optimization algorithms such as genetic algorithm (GA), differential evolution (DE), evolutionary strategies (ES), and particle swarm optimization (PSO) algorithms [16–18]. Nevertheless, ABC has been found to suffer from few limitations such as slow convergence speed [19, 20] and premature convergence [21, 22]. Due to that, researchers have tried to solve them by developing various ABC variants, for example, Gbest-guided ABC (GABC) by Zhu and Kwong in 2010 [23], Best-so-far ABC (BsfABC) by Banharnsakun et al. [24], and Improved ABC (IABC) by Gao and Liu [25] in 2011 as well as modified ABC (MABC) by Gao and Liu [20], Global-best ABC (BABC) by Gao et al. [19], and enhanced ABC by Abro and Mohamad-Saleh [26] in 2012. However, some of these variants are still incapable of efficiently solving the problems, whilst a number of the variants could still be improved. For instance, the idea of IABC using the best solution is very convincing because it enhances the convergence speed [25]. Furthermore, its incorporation of random search equation into the algorithm is rather promising as the equation is known for its randomness and able to generate diverse population [25]. However, IABC is unable to solve Rosenbrock function as it is actually poor in exploitation [25]. Meanwhile, one of the BABC variants, BABC1, has also incorporated the idea of using the previous best solution as the guidance for the search [19]. With some adjustment to the solution search equation, BABC1 has shown the best performance among other variants at that time. Nevertheless, BABC1 is actually prone to premature convergence when dealing with complex multimodal problems [27]. With the motivation from one of the BABC variants which is BABC2, enhanced ABC (EABC) has been proposed with the idea to balance the exploration and exploitation abilities of the algorithm. Nonetheless, EABC has a tendency to suffer from slow convergence speed (i.e., lack of exploitation process) as shown in [28]. With the motivation from the existing ABC variants and their limitations, a new modified ABC is proposed in this paper. This new enhanced ABC is expected to give excellent performance in terms of convergence speed and robust global minimum search.

## 2. Artificial Bee Colony (ABC) Algorithm Model

**Figure 1:**
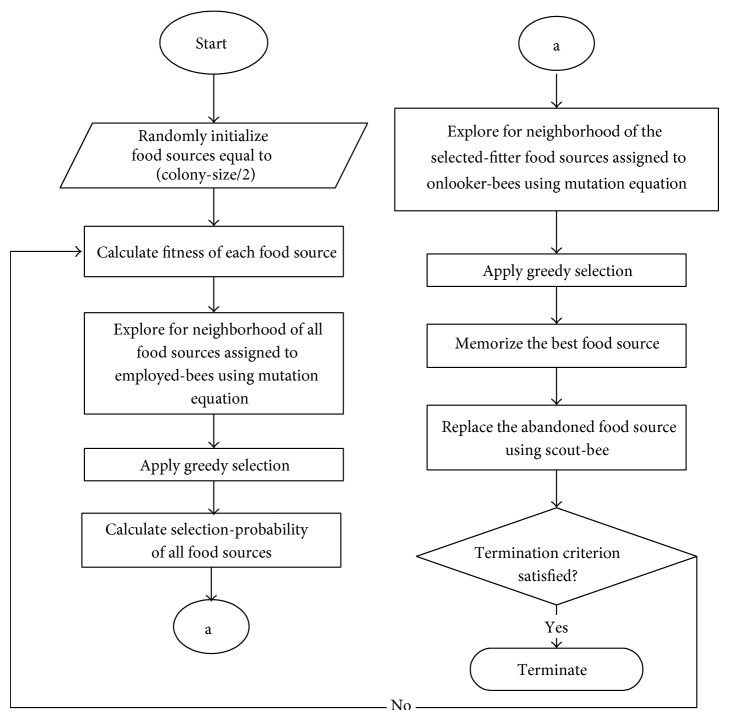
The flowchart of standard ABC algorithm.

The standard ABC algorithm is a population-based optimization algorithm. The working principle of ABC is as illustrated in Figure 1. Based on the figure, the working principle of ABC can be categorized into five main phases which are initialization, employed-bees, onlooker-bees, scout-bee, and termination phases which consist of a total of twelve stages or processes.

In ABC, three phases are performance-deciding phases which are employed-bees, onlooker-bees, and scout-bee phases while the other two are supporting phases. The exploration process of the algorithm takes place in employed-bees and onlooker-bees phases where the bees need to explore the neighborhood of the food sources allocated to them. Meanwhile, the exploitation process happens in the onlooker-bees phase when onlooker-bees apply fitness-proportion selection scheme in order to select the selected-fitter food sources. The details of the phases are discussed in the following subsections and more details of ABC can also be found in [18].

### 2.1. Initialization

In ABC algorithm, food sources represent the possible solution among the population of a problem. They are randomly initialized. The initialization of the population is based on user predetermined values of the population size. These food sources are then assigned to the employed-bees. Next, the nectar amounts which represent the fitness value of each food source are calculated using equation found in [18, 29, 30]:

(1) $$\begin{array}{c} \text{fi}{\text{t}}_{i}=\left\{\begin{array}{cc} \frac{1}{1+f_{i}}, & f_{i}\geq 0,\\ 1+\text{abs}\left(f_{i}\right), & f_{i}<0, \end{array}\right. \end{array}$$

where *f*_*i*_ is objective function value of *i*th food source.

### 2.2. Employed-Bees Phase

In this phase, employed-bees explore the neighborhood of the food sources assigned to them and update the food sources using the mutation equation given by

(2) $$\begin{array}{c} z_{ij}=y_{ij}+\phi_{ij}\left(y_{ij}-y_{kj}\right), \end{array}$$

where *z*_*ij*_ is the candidate solution of food sources, *y*_*ij*_ is the *j*th dimension of the *i*th food sources, and *y*_*kj*_ is the *k*th food sources that are randomly chosen from a neighborhood of *i*th food sources for *k* ∈ [1,2,…, *SN*] and *SN* is the number of food sources. Subscripts *k* and *i* are mutually exclusive food sources. For the equation, *k* and *j* are chosen randomly and *j* ∈ [1,2,…, *D*] where *D* represents the dimension of the search space and *ϕ*_*ij*_ is the control parameter that represents random number from [−1,1], inclusively.

The explorations by employed-bees generate new food sources (i.e., candidate solutions of food sources). A selection between the candidate solution and the old food sources is based on which of them exhibits the best fitness value. This selection is done using greedy-selection scheme. The chosen food sources are potentially fitter food sources and are shared with onlooker-bees in onlooker-bees phase.

### 2.3. Onlooker-Bees Phase

During this phase, the onlooker-bees do not update all potentially fitter food sources shared with them by employed-bees. They apply fitness-proportion selection scheme to choose few selected-fitter food sources among all the food sources shared with them. The exploitation of the food sources by onlooker-bees has actually made the algorithm converge fast. The fitness-proportion selection scheme is dependent on the probability value, *P*_*i*_ given by

(3) $$\begin{array}{c} P_{i}=\frac{\text{fi}{\text{t}}_{i}}{\sum_{j=1}^{SN}{\text{fit}}_{j}}, \end{array}$$

where *P*_*i*_ is the probability of *i*th food source, fit_*i*_ is the fitness value of *i*th food source, and *SN* represents the number of available food sources.

Onlooker-bees then explored the neighborhood of the selected-fitter food sources and update the food sources using the equation given in (2). The new candidate solution is then compared with the old food source using the greedy-selection scheme. Next, the best food source so far for that generation is memorized before entering the scout-bee phase.

### 2.4. Scout-Bee Phase

In scout-bee phase, a food source which has become exhausted and does not show improvement over a* limit* is abandoned [23].* Limit* is a control parameter used to signify exhausted food source [19]. Employed-bee whose food source has reached* limit* will become scout-bee. The scout-bee will take consequent flights and search the search space randomly to find new food source using

(4) $$\begin{array}{c} y_{i}^{j}=y_{\min}^{j}+\text{rand}\left(0,1\right)\left(y_{\max}^{j}-y_{\min}^{j}\right), \end{array}$$

where *y*_min⁡_^*j*^ and *y*_max⁡_^*j*^ are the lower and upper limit of the search space, respectively. rand(0,1) is a function which randomly generates numbers within [0,1]. This action is necessary for the scout-bee to replace the abandoned food source with new food source and thus balance the number of populations again.

### 2.5. Termination

The termination criterion of the algorithm is based on the maximum number of generations or maximum cycle number (MCN) [18]. This number is preset by user prior to the simulation of ABC algorithm.

## 3. New Enhanced ABC (JA-ABC5) Algorithm

**Figure 2:**
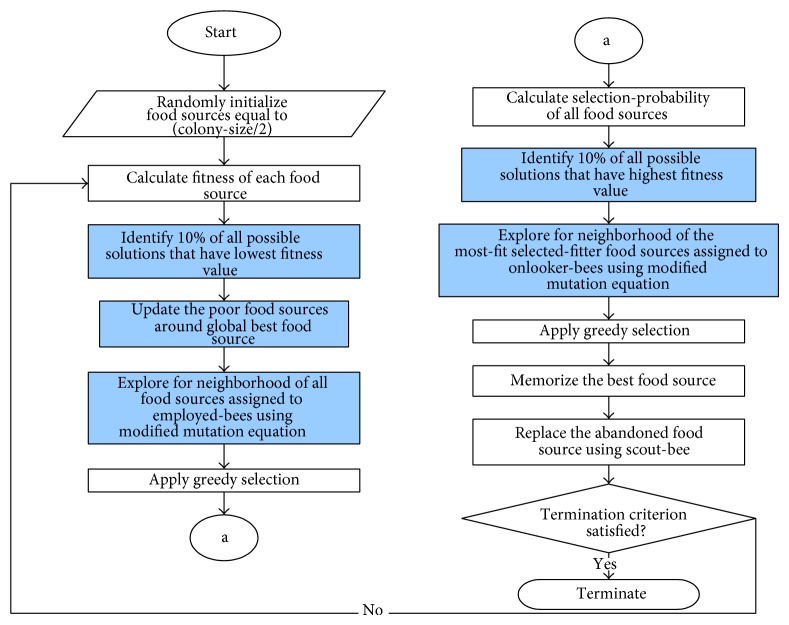
The flowchart of new enhanced ABC (JA-ABC5) algorithm.

The limitations of ABC are due to (2) that is known to be good in exploration but poor in exploitation. This imbalances of exploration and exploitation capabilities of the standard ABC algorithm contribute to its lack in performance. Thus, few modifications have been introduced to the standard ABC algorithm for the purpose of balancing the exploration and exploitation capabilities of the algorithm. The proposed algorithm introduces four modifications to the standard ABC algorithm as highlighted in Figure 2.

The first modification is the insertion of new phase between initialization and employed-bees phases. This phase consists of two stages illustrated by stages 4 and 5 in Figure 2. The first stage aims to identify few food sources that have the lowest fitness values, referred to as poor food sources. Next, these poor food sources are updated around global best (*g*-best) food source using the mutation equation inspired from [19] given by

(5) $$\begin{array}{c} z_{ij}=y_{\text{best},j}+\phi_{ij}\left(y_{pj}-y_{kj}\right), \end{array}$$

where *z*_*ij*_ represents the candidate solution of *i*th food source with *j*th dimension. *y*_best,*j*_ is the best food source, *y*_*pj*_ is *j*th dimension of *p*th food source and is randomly chosen. Subscripts *i*, *k*, and *p* are mutually exclusive food sources and the rest of the parameters are the same as in (2).

The generated food sources would now be fitter since they are being directed towards the global best food source based on (5). This has increased the exploitation process of the algorithm and makes the current population consist of fitter food sources. The random selection of food sources has also made the population not only fitter, but diverse as well.

Then, in employed-bees phases, the fitter populations are updated. Here comes the second modification which is represented by stage 6 in Figure 2. Since the population is now fitter, there is a possibility for the algorithm to be trapped in local optima. Thus, to overcome this, the exploration process should be enhanced. The enhancement of the exploration process has been done by adapting new mutation equation in employed-bees phase. This new mutation equation is obtained by adapting modified mutation equation inspired from [25] which is well known for its randomness. The modification produces a modified equation given by

(6) $$\begin{array}{c} z_{ij}=y_{r1j}+\phi_{ij}\left(y_{r2j}-y_{r3j}\right), \end{array}$$

where *z*_*ij*_ represents the candidate solution of *i*th food source with *j*th dimension. *y*_*r*1*j*_, *y*_*r*2*j*_, and *y*_*r*3*j*_ are the *r*1th, *r*2th, and *r*3th food sources that are randomly chosen from neighborhood of *i*th food sources. Subscripts *r*1, *r*2, and *r*3 are mutually exclusive food sources and the rest of the parameters are the same as in (2). Equation (6) updates the food sources by directing the interaction among randomly chosen food sources. This increases the diversity of the exploration process that enhances the capability of the algorithm to avoid local optima trapping.

The next modification is aimed at increasing the convergence speed of the algorithm since random searching has a tendency to slow down the execution of the algorithm. The enhancement of the exploitation capability in onlooker-bees phase has been formulated to overcome this problem. The onlooker-bees have been directed to update only few most-fit-selected-fitter food sources. As already mentioned, onlooker-bees basically do not update all food sources but update only selected-fitter food sources. Hence, in this proposed algorithm, onlooker-bees will update only few most-fit food sources among the selected-fitter food sources. Thus, with only few fitter food sources to be updated, the convergence speed of the algorithm has been increased. This modification is shown by stage 9 in Figure 2.

The fourth modification is to replace the mutation of onlooker-bees from (2) to the equation adapted from the work of [25]

(7) $$\begin{array}{c} z_{ij}=y_{\text{best},j}+\phi_{ij}\left(y_{ij}-y_{mj}\right), \end{array}$$

where *z*_*ij*_ represents the candidate solution of *i*th food source with *j*th dimension. *y*_best,*j*_ is the best food source and *y*_*mj*_ represents *j*th dimension of *m*th food source and is randomly chosen. Subscripts *i* and *m* are mutually exclusive food sources and the rest of the parameters are the same as (2).

Equation (7) is able to enhance the convergence speed since the fitter food sources in onlooker-bees phase have been updated towards the *g*-best food sources. This modification is presented by stage 10 in Figure 2. Thus, in the end, the proposed algorithm, JA-ABC5, has enhanced and balanced exploration and exploitation processes. With this, it is expected to converge faster and to be able to reach global optimum efficiently. Its ability is assessed by comparing its performance with existing variants on 27 benchmark functions and at solving the reactive power optimization problem.

## 4. Simulations on Benchmark Functions

In order to justify the robustness of the proposed JA-ABC5 algorithm, it has been simulated on 27 commonly used benchmark functions as listed in Table 1. These benchmark functions vary from different types of functions such as random shifted, unimodal, multimodal, and rotated functions prior to testing the capabilities of the algorithm to solve a wide range of problems.

The performance of JA-ABC5 has been compared with the standard ABC algorithm and three other sophisticated existing ABC variants: Improved ABC (IABC) [25], Global best ABC (BABC1) [19], and enhanced ABC (EABC) [26, 29] to show the effectiveness of JA-ABC5 in solving those functions.

For all algorithms, the dimensionality of the benchmark functions has been set to 30, the population size has been set to 50, number of generations has been limited to 1000, and the parameter* limit* has been set as *D* × *SN*, where *D* represents the dimension of the search space and *SN* is the number of food sources. The *P value* of IABC has been set to 0.25 [25]. As for global solution validation, each of the compared algorithms including JA-ABC5 has been set to be simulated for 30 times on each benchmark function [26]. All these values follow those used and recommended in the literature [18–20, 23, 25, 26, 30].

The simulation and testing process have been carried out using Matlab R2010a on an Intel Core i7 with 2.80 GHz speed computer.

### 4.1. Results of Benchmark Functions Simulation

**Figure 3:**
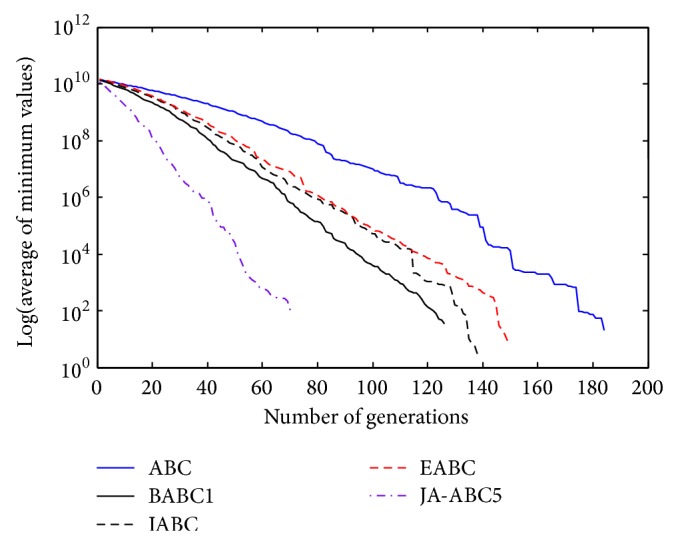
The convergence rates of optimization algorithms on Himmelblau function.

**Figure 4:**
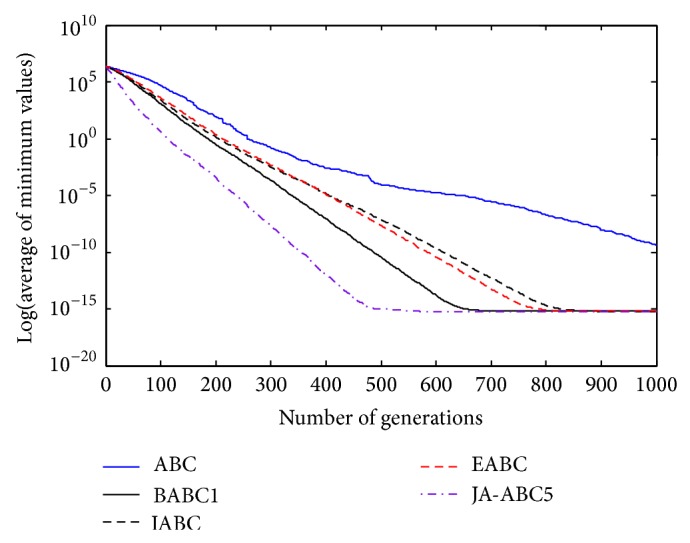
The convergence rates of optimization algorithms on Random Shifted Sphere function.

**Figure 5:**
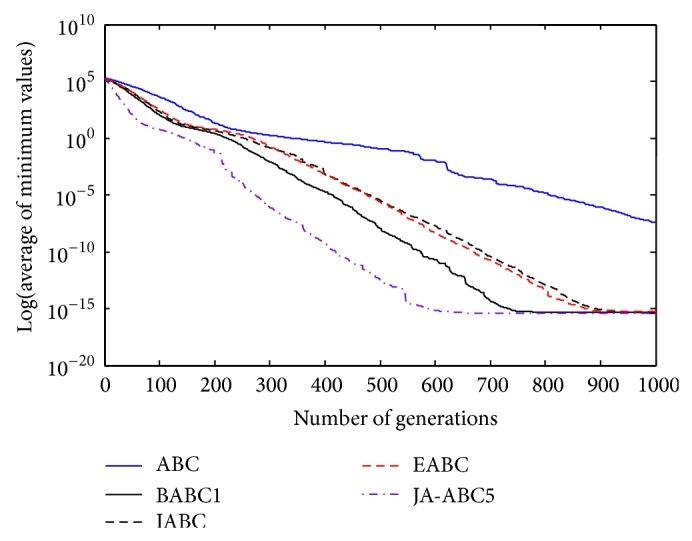
The convergence rates of optimization algorithms on Bohachevsky 2 function.

**Figure 6:**
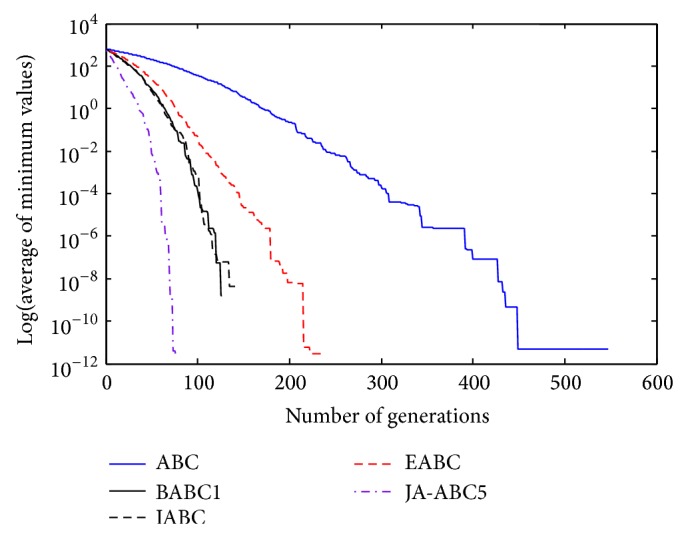
The convergence rates of the optimization algorithms on Rotated Griewank function.

**Figure 7:**
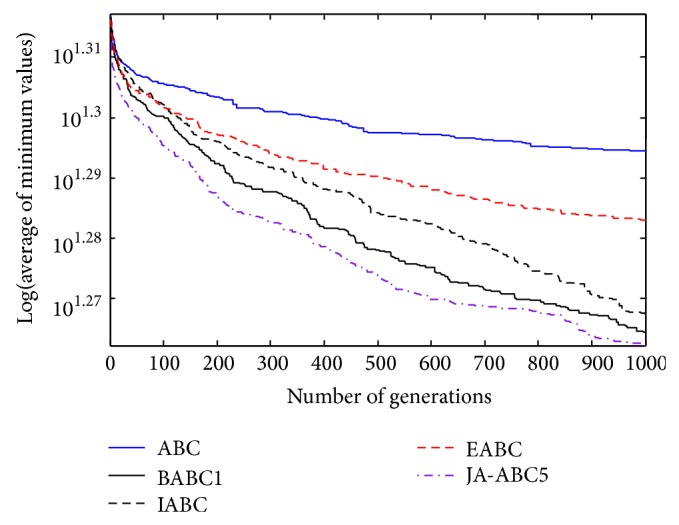
The convergence rates of the optimization algorithms on Rotated Ackley function.

Figures 3, 4, 5, 6, and 7 show the graphical results of the proposed algorithm, JA-ABC5 algorithm. The figures have shown that the proposed algorithm has outperformed other algorithms in terms of convergence speed. It exhibits faster convergence as compared to others. Moreover, the considerable difference of the proposed algorithm in comparison with other compared variants has clearly justified that the proposed algorithm is a robust ABC variant that has potential to solve optimization problems. The standard ABC exhibits the worst performance among all since it has suffered from few limitations as mentioned earlier.

Meanwhile, the statistical data in Table 2 reveal the numerical performance results of various ABC variants illustrating the values of minimum, mean, and standard deviation of the compared optimization algorithms. The results have shown that JA-ABC5 exhibits the least value of minimum, mean, and standard deviation on most of the benchmark functions. Thus, this vividly demonstrates that JA-ABC5 has the best performance in comparison with other compared ABC variants.

## 5. Reactive Power Optimization Application

Reactive power optimization (RPO) is known to be a large-scale nonlinear combinatorial constrained problem [31]. RPO basically serves to determine the optimal setting of the power system network to satisfy few constraints such as the power flow equation system security and equipment operating limits [32]. This problem has been discovered by Carpentier in 1962 [33] and, since then, many have tried to solve it. Researchers and engineers have tried to solve it by developing various search strategies since this kind of problem is very essential to be solved. This is because this problem is the important tool in the power system's operation and planning [34] since it actually has close contact with the security and economic dispatch of a power system [35]. For example, they have attempted to solve RPO problem using various classical methods such as linear programming, Newton method, interior point, and many more. Nonetheless, the methods have shown some inefficiency in solving it [31]. Recently, researchers have tried to implement stochastic and heuristics techniques to solve this problem [31]. Thus, this has shown that RPO basically can be a perfect tool in order to validate the robustness of the proposed algorithm.

RPO problem is a combinatorial nonlinear constrained problem. The general mathematical formulation for that kind of problem is given by

(8) $$\begin{array}{c} \min\left(f\left(x\right)\right) \end{array}$$

such that

(9) g(x)=0,h(x)≤0,

where *f*(*x*) is the objective function to be minimized, *g*(*x*) is the equality constraints, and *h*(*x*) is the inequality constraints. Hence, the mathematical formulation of RPO problem with equality and inequality constraints is discussed in next subsections.

### 5.1. Objective Function-Active Power Loss

The objective function for RPO problem can be either the active power loss, total cost of compensation, total energy generation cost, and many more [31]. In this paper, only active power loss is considered as the objective function to be solved by the proposed algorithm, JA-ABC5. The mathematical formulation of active power loss is given by

(10) Ploss=∑k=1NLPloss,k=∑i=1N∑j=1NgijVi2+Vj2−2ViVjcos⁡θij,

where *P*_loss_ is an active power loss, *g*_*ij*_ is the conductance between bus *i* and bus *j*, *V*_*i*_ is the voltage magnitude of bus *i*, *V*_*j*_ is the voltage magnitude of bus *j*, *θ*_*ij*_ is the angle difference of *ij*th transmission line, *N* is the total number of system's buses, and *NL* is the total number of transmission lines.

### 5.2. Equality Constraints

The equality constraints of the problem has been set to the power flow equations given by [36]

(11) $$\begin{array}{c} P_{Gi}-P_{Di}-\overset{N}{\underset{j=1}{\sum }}\left|V_{i}\right|\left|V_{j}\right|\left|Y_{ij}\right|\cos\left(\theta_{ij}-\delta_{i}+\delta_{j}\right)=0,\\ Q_{Gi}-Q_{Di}+\overset{N}{\underset{j=1}{\sum }}\left|V_{i}\right|\left|V_{j}\right|\left|Y_{ij}\right|\sin\left(\theta_{ij}-\delta_{i}+\delta_{j}\right)=0, \end{array}$$

where *P*_*Gi*_ is the active power generation at bus *i*, *P*_*Di*_ is the active power demand at bus *i*, *Q*_*Gi*_ is the reactive power generation at bus *i*, *Q*_*Di*_ is the reactive power demand at bus *i*, *Y*_*ij*_ is the admittance between bus *i* and bus *j*, *δi* and *δj* are the voltage angle at bus *i* and bus *j*, respectively, and the rest of the parameters are the same as in (10).

### 5.3. Inequality Constraints

The inequality constraints of the problem are the control variables that are to be optimized within their ranges. These control variables are the food sources or possible solutions that need to be optimized by JA-ABC5. The range of the possible solutions follows the following limits:

(12) $$\begin{array}{c} P_{Gi}^{\min}\leq P_{Gi}\leq P_{Gi}^{\max},\\ V_{i}^{\min}\leq V_{i}\leq V_{i}^{\max},\\ Q_{Ci}^{\min}\leq Q_{Ci}\leq Q_{Ci}^{\max},\\ T_{i}^{\min}\leq T_{i}\leq T_{i}^{\max}, \end{array}$$

where *P*_*Gi*_ is the active power generation at bus *i*, *V*_*i*_ is the voltage magnitude at bus *i*, *Q*_*Ci*_ is the shunt compensation at bus *i*, and *T*_*i*_ is the transformer tap setting at bus *i*. Moreover, *P*_*Gi*_^min⁡^ and *P*_*Gi*_^max⁡^ are lower and upper limits of active power generation, *V*_*i*_^min⁡^ and *V*_*i*_^max⁡^ are lower and upper limits of voltage magnitude, *Q*_*Ci*_^min⁡^ and *Q*_*Ci*_^max⁡^ are lower and upper limits of shunt compensation, and *T*_*i*_^min⁡^ and *T*_*i*_^max⁡^ are lower and upper limits of tap setting.

### 5.4. Penalty Function

Penalty function is derived in order to convert constrained problem to unconstrained problem by adding penalty terms. Since RPO problem consists of several constraints as mentioned in the previous subsection, penalty terms have been added to (10) and the equation for the objective function of the problem now becomes

(13) $$\begin{array}{c} P\left(x\right)=P_{\text{loss}}+\Omega_{P}+\Omega_{Q}+\Omega_{V}+\Omega_{G}+\Omega_{C}+\Omega_{T}, \end{array}$$

where *P*(*x*) is the penalty function and Ω_*P*_, Ω_*Q*_, Ω_*V*_, Ω_*G*_, Ω_*C*_, and Ω_*T*_ are the penalty terms of the listed equality and inequality constraints, respectively. Thus, the penalty terms are given by

(14) ΩP=ρ∑i=1NPGi−PDi−Vi∑i=1NVjgijsinθij+Bijcos⁡θij2,ΩQ=ρ∑i=1NQGi+QCi−QDiVi∑i=1NVjgijsinθij−Bijcos⁡θijρ∑i=1N5.−Vi∑i=1NVjgijsinθij−Bijcos⁡θij2,ΩV=ρ∑i=1Nmax⁡0,Vi−Vimax⁡2+ρ∑i=1Nmax⁡0,Vimin⁡−Vi2,ΩG=ρ∑i=1NGmax⁡0,PGi−PGimax⁡2+ρ∑i=1NGmax⁡0,PGimin⁡−PGi2,ΩC=ρ∑i=1NCmax⁡0,QCi−QCimax⁡2+ρ∑i=1NCmax⁡0,QCimin⁡−QCi2,ΩT=ρ∑i=1NTmax⁡0,Ti−Timax⁡2+ρ∑i=1NTmax⁡0,Timin⁡−Ti2,

where *P*_*Gi*_ is the active power generation at bus *i*, *P*_*Di*_ is the active power demand at bus *i*, *Q*_*Gi*_ is the reactive power generation at bus *i*, *Q*_*Di*_ is the reactive power demand at bus *i*, *Q*_*Ci*_ is the shunt compensation at bus *i*, *T*_*i*_ is the transformer tap settings of transformer *i*, *B*_*ij*_ is the susceptance between bus *i* and bus *j*, *NG* is the total number of generators, *NC* is the total number of shunt compensator, *NT* is the total number of transformers, and the rest of the parameters are the same as in (10) [31, 36].

The proposed algorithm, JA-ABC5, is implemented to solve RPO problem by finding the optimal possible solutions to solve the objective function which is the penalty function obtained from (13). The possible solutions that need to be optimized which basically act as the food sources of JA-ABC5 are given by the previous subsection. They are active power generation, *P*_*G*_, voltage magnitude, *V*, shunt compensation, *Q*_*C*_, and transformer tap setting, *T*, at the required bus. JA-ABC5 is expected to produce less value of power loss which is affected by the above mentioned control variables' values. Thus, it is important to find the optimal values or settings of the control variables so that less amount of power loss has been generated.

### 5.5. Results of RPO

For the purpose of solving the RPO problem, IEEE 30-bus power system data has been obtained from [31]. To validate the performance of JA-ABC5 in solving the RPO problem, it has been compared with three existing ABC variants: IABC [25], BABC1 [19], and EABC [26, 29] as well as with other optimization algorithms available in the work of [31] which are self-adaptive real coded genetic algorithm (SARGA) [37], particle swarm optimization (PSO) [38], comprehensive learning PSO (CLPSO) [38], and enhanced genetic algorithm with decoupled quadratic load flow (EGA-DQLF) [39]. The performance of JA-ABC5 in solving the RPO problem in comparison with other optimization algorithms is tabulated in Table 3.

From Table 3, it is clear that variants of ABC algorithm have outperformed the other optimization algorithms. Most importantly, the results have shown that the proposed algorithm, JA-ABC5, has produced the minimum power loss of 1.4985 MW when compared to other optimization algorithms. Thus, this vividly shows that JA-ABC5 is able to solve complex optimization problem and hence can be applied to solve other optimization problems.

## 6. Conclusion

This work presents a new variant of the ABC algorithm referred to as JA-ABC5 by modifying the standard ABC algorithm to balance out the effects of exploration and exploitation processes into the performance of the algorithm. The balanced exploration and exploitation capabilities are able to enhance the performance of the algorithm in terms of convergence speed and global optimum achievement. The performance results have clearly exhibited the best performance of JA-ABC5 in comparison to the compared ABC variants on 27 benchmark functions. Moreover, the efficiency of the algorithm in solving a complex real-world problem, the reactive power optimization (RPO), has vividly depicted that the algorithm is robust, effective, and reliable in solving optimization problems.

## Figures and Tables

**Table 1:** Benchmark functions.

Function	Function name	Initialization range
*f*1	Griewank	*±600 *
*f*2	Rastrigin	*±15 *
*f*3	Rosenbrock	*±15 *
*f*4	RS Ackley	*±32 *
*f*5	Schwefel	*±500 *
*f*6	Himmelblau	*±600 *
*f*7	RS Sphere	*±600 *
*f*8	Step	*±600 *
*f*9	Bohachevsky 2	*±100 *
*f*10	RS Schwefel 2.22	*±100 *
*f*11	RS Schwefel Ridges	*±100 *
*f*12	RS Schwefel Ridges with Noise	*±15 *
*f*13	RS Elliptic	*±100 *
*f*14	Zekhelip	*±15 *
*f*15	Non-continuous Rastrigin	*±15 *
*f*16	Michalewicz	*0*–*180 *
*f*17	First Expanded Function	*±15 *
*f*18	Second Expanded Function	*±15 *
*f*19	Third Expanded Function	*±15 *
*f*20	Fourth Expanded Function	*±500 *
*f*21	Fifth Expanded Function	*±100 *
*f*22	Sixth Expanded Function	*±100 *
*f*23	Seventh Expanded Function	*±15 *
*f*24	Eighth Expanded Function	*±100 *
*f*25	Rotated Griewank Function	*0*–*600 *
*f*26	Rotated Ackley Function	*±32 *
*f*27	Rotated Rastrigin Function	*±5 *

**Table 2:** Statistical Results of Optimization Algorithms.


*f*1	MIN	Average	STD DEV	*f*15	MIN	Average	STD DEV

ABC	1.22*E* − 14	1.47*E* − 13	1.65*E* − 13	ABC	3.56*E* − 07	9.44*E* − 01	8.38*E* − 01
BABC1	4.44*E* − 16	6.22*E* − 16	1.50*E* − 16	BABC1	0.00*E* + 00	2.00*E* − 01	4.84*E* − 01
IABC	4.44*E* − 16	6.40*E* − 16	4.82*E* − 16	IABC	0.00*E* + 00	7.01*E* − 14	6.95*E* − 14
EABC	4.44*E* − 16	6.31*E* − 16	1.58*E* − 16	EABC	0.00*E* + 00	6.72*E* − 12	2.10*E* − 11
JA-ABC5	3.33*E* − 16	5.48*E* − 16	8.71*E* − 17	JA-ABC5	0.00*E* + 00	1.00*E* − 01	3.05*E* − 01

*f*2	MIN	Average	STD DEV	*f*16	MIN	Average	STD DEV

ABC	2.29*E* − 08	3.77*E* − 01	5.87*E* − 01	ABC	−2.92*E* + 01	−2.90*E* + 01	1.40*E* − 01
BABC1	0.00*E* + 00	2.98*E* − 01	4.64*E* − 01	BABC1	−2.96*E* + 01	−2.94*E* + 01	1.08*E* − 01
IABC	0.00*E* + 00	0.00*E* + 00	0.00*E* + 00	IABC	−2.96*E* + 01	−2.96*E* + 01	1.58*E* − 02
EABC	0.00*E* + 00	8.02*E* − 14	3.02*E* − 13	EABC	−2.96*E* + 01	−2.95*E* + 01	9.68*E* − 02
JA-ABC5	0.00*E* + 00	2.32*E* − 01	5.01*E* − 01	JA-ABC5	−2.96*E* + 01	−2.90*E* + 01	1.29*E* − 02

*f*3	MIN	Average	STD DEV	*f*17	MIN	Average	STD DEV

ABC	2.23*E* − 02	9.03*E* + 00	2.11*E* + 00	ABC	7.85*E* − 03	4.09*E* − 02	1.81*E* − 01
BABC1	2.32*E* − 02	3.04*E* + 01	3.30*E* + 01	BABC1	7.85*E* − 03	5.69*E* − 01	5.62*E* − 01
IABC	9.66*E* − 02	5.63*E* + 00	5.85*E* + 00	IABC	7.85*E* − 03	4.08*E* − 02	1.81*E* − 01
EABC	1.48*E* − 02	4.42*E* + 00	7.16*E* + 00	EABC	7.85*E* − 03	7.85*E* − 03	1.75*E* − 16
JA-ABC5	1.31*E* − 02	4.97*E* + 00	1.44*E* + 01	JA-ABC5	3.03*E* − 03	6.55*E* − 03	1.37*E* − 01

*f*4	MIN	Average	STD DEV	*f*18	MIN	Average	STD DEV

ABC	2.48*E* − 06	1.82*E* − 05	1.12*E* − 05	ABC	1.59*E* − 14	3.29*E* − 13	2.53*E* − 13
BABC1	3.46*E* − 14	4.86*E* − 14	7.52*E* − 15	BABC1	4.42*E* − 16	6.29*E* − 16	1.05*E* − 16
IABC	8.98*E* − 12	1.97*E* − 11	6.75*E* − 12	IABC	4.44*E* − 16	5.72*E* − 16	8.91*E* − 17
EABC	1.66*E* − 12	4.15*E* − 12	1.56*E* − 12	EABC	4.92*E* − 16	6.62*E* − 16	1.23*E* − 16
JA-ABC5	2.75*E* − 14	3.12*E* − 14	3.16*E* − 15	JA-ABC5	4.19*E* − 16	5.32*E* − 16	7.03*E* − 17

*f*5	MIN	Average	STD DEV	*f*19	MIN	Average	STD DEV

ABC	1.07*E* − 01	2.96*E* + 02	1.20*E* + 02	ABC	2.80*E* + 01	3.13*E* + 01	6.05*E* + 00
BABC1	3.82*E* − 04	1.54*E* + 02	1.33*E* + 02	BABC1	2.78*E* + 01	6.29*E* + 01	3.13*E* + 01
IABC	3.82*E* − 04	6.71*E* + 01	8.62*E* + 01	IABC	2.80*E* + 01	4.20*E* + 01	1.87*E* + 01
EABC	3.82*E* − 04	9.28*E* + 01	1.06*E* + 02	EABC	2.76*E* + 01	4.76*E* + 01	2.68*E* + 01
JA-ABC5	3.82*E* − 04	1.03*E* + 02	1.23*E* + 02	JA-ABC5	2.74*E* + 01	4.18*E* + 01	1.30*E* + 01

*f*6	MIN	Average	STD DEV	*f*20	MIN	Average	STD DEV

ABC	−7.83*E* + 01	−7.83*E* + 01	1.55*E* − 07	ABC	3.20*E* − 08	6.65*E* − 02	3.64*E* − 01
BABC1	−7.83*E* + 01	−7.82*E* + 01	4.09*E* − 01	BABC1	0.00*E* + 00	5.98*E* − 01	9.30*E* − 01
IABC	−7.83*E* + 01	−7.83*E* + 01	1.21*E* − 14	IABC	0.00*E* + 00	0.00*E* + 00	0.00*E* + 00
EABC	−7.83*E* + 01	−7.83*E* + 01	2.28*E* − 01	EABC	0.00*E* + 00	1.67*E* − 16	5.18*E* − 16
JA-ABC5	−7.83*E* + 01	−7.83*E* + 01	2.39*E* − 01	JA-ABC5	0.00*E* + 00	1.33*E* − 01	5.06*E* − 01

*f*7	MIN	Average	STD DEV	*f*21	MIN	Average	STD DEV

ABC	4.60*E* − 11	4.53*E* − 10	3.99*E* − 10	ABC	1.66*E* − 10	2.30*E* − 09	3.28*E* − 09
BABC1	4.55*E* − 16	6.66*E* − 16	1.10*E* − 16	BABC1	1.11*E* − 16	3.89*E* − 16	1.66*E* − 16
IABC	4.09*E* − 16	5.89*E* − 16	9.03*E* − 16	IABC	5.55*E* − 17	3.11*E* − 16	1.85*E* − 16
EABC	4.72*E* − 16	6.57*E* − 16	1.24*E* − 16	EABC	1.11*E* − 16	4.86*E* − 16	1.32*E* − 16
JA-ABC5	3.29*E* − 16	5.62*E* − 16	1.08*E* − 16	JA-ABC5	5.55*E* − 17	3.05*E* − 16	1.21*E* − 16

*f*8	MIN	Average	STD DEV	*f*22	MIN	Average	STD DEV

ABC	4.27*E* − 11	4.82*E* − 10	5.12*E* − 10	ABC	1.80*E* − 10	2.89*E* − 09	3.60*E* − 09
BABC1	4.54*E* − 16	6.37*E* − 16	1.02*E* − 16	BABC1	0.00*E* + 00	3.94*E* − 16	1.45*E* − 16
IABC	2.87*E* − 16	5.38*E* − 16	9.62*E* − 17	IABC	0.00*E* + 00	2.55*E* − 16	1.30*E* − 16
EABC	4.69*E* − 16	6.65*E* − 16	8.62*E* − 17	EABC	2.22*E* − 16	4.37*E* − 16	1.33*E* − 16
JA-ABC5	4.21*E* − 16	4.66*E* − 16	7.66*E* − 17	JA-ABC5	0.00*E* + 00	2.46*E* − 16	1.28*E* − 16

*f*9	MIN	Average	STD DEV	*f*23	MIN	Average	STD DEV

ABC	9.72*E* − 10	3.85*E* − 08	4.32*E* − 08	ABC	7.05*E* − 12	1.60*E* − 10	1.70*E* − 10
BABC1	1.11*E* − 16	4.57*E* − 16	1.36*E* − 16	BABC1	4.32*E* − 16	6.20*E* − 16	1.07*E* − 16
IABC	1.11*E* − 16	4.09*E* − 16	1.42*E* − 16	IABC	3.23*E* − 16	5.57*E* − 16	8.87*E* − 17
EABC	2.22*E* − 16	5.41*E* − 16	1.76*E* − 16	EABC	4.80*E* − 16	6.78*E* − 16	9.50*E* − 17
JA-ABC5	1.11*E* − 16	3.81*E* − 16	1.27*E* − 16	JA-ABC5	3.22*E* − 16	5.57*E* − 16	8.72*E* − 17

*f*10	MIN	Average	STD DEV	*f*24	MIN	Average	STD DEV

ABC	1.96*E* − 06	1.07*E* − 05	5.44*E* − 06	ABC	7.50*E* − 01	7.50*E* − 01	2.20*E* − 04
BABC1	5.55*E* − 17	2.44*E* − 15	1.79*E* − 15	BABC1	7.50*E* − 01	7.50*E* − 01	3.23*E* − 16
IABC	6.93*E* − 12	2.64*E* − 11	9.63*E* − 12	IABC	7.50*E* − 01	7.50*E* − 01	3.44*E* − 16
EABC	2.95*E* − 12	8.20*E* − 12	4.41*E* − 12	EABC	7.50*E* − 01	7.50*E* − 01	1.78*E* − 16
JA-ABC5	0.00*E* + 00	1.85*E* − 18	1.01*E* − 17	JA-ABC5	7.50*E* − 01	7.50*E* − 01	1.36*E* − 16

*f*11	MIN	Average	STD DEV	*f*25	MIN	Average	STD DEV

ABC	1.85*E* − 10	2.62*E* − 09	2.40*E* − 09	ABC	0.00*E* + 00	0.00*E* + 00	0.00*E* + 00
BABC1	3.93*E* − 16	6.42*E* − 16	1.11*E* − 16	BABC1	0.00*E* + 00	0.00*E* + 00	0.00*E* + 00
IABC	3.95*E* − 16	5.77*E* − 16	9.15*E* − 17	IABC	0.00*E* + 00	0.00*E* + 00	0.00*E* + 00
EABC	4.39*E* − 16	6.54*E* − 16	1.04*E* − 16	EABC	0.00*E* + 00	0.00*E* + 00	0.00*E* + 00
JA-ABC5	3.31*E* − 16	5.76*E* − 16	1.06*E* − 17	JA-ABC5	0.00*E* + 00	0.00*E* + 00	0.00*E* + 00

*f*12	MIN	Average	STD DEV	*f*26	MIN	Average	STD DEV

ABC	3.33*E* − 03	8.34*E* − 03	3.92*E* − 03	ABC	1.79*E* + 01	1.97*E* + 01	4.65*E* − 01
BABC1	4.60*E* − 13	1.77*E* − 12	1.30*E* − 12	BABC1	1.70*E* + 01	1.82*E* + 01	6.89*E* − 01
IABC	5.65*E* − 09	1.82*E* − 08	1.15*E* − 08	IABC	1.66*E* + 01	1.85*E* + 01	7.64*E* − 01
EABC	2.50*E* − 10	8.02*E* − 10	5.34*E* − 10	EABC	1.80*E* + 01	1.92*E* + 01	5.92*E* − 01
JA-ABC5	5.24*E* − 16	8.08*E* − 16	1.71*E* − 16	JA-ABC5	1.60*E* + 01	1.73*E* + 01	3.86*E* − 01

*f*13	MIN	Average	STD DEV	*f*27	MIN	Average	STD DEV

ABC	1.63*E* − 10	7.50*E* − 09	1.60*E* − 08	ABC	2.98*E* + 02	3.25*E* + 02	1.36*E* + 01
BABC1	4.49*E* − 16	6.38*E* − 16	1.06*E* − 16	BABC1	2.12*E* + 02	2.88*E* + 02	2.01*E* + 01
IABC	4.21*E* − 16	5.41*E* − 16	9.08*E* − 17	IABC	2.59*E* + 02	2.99*E* + 02	1.97*E* + 01
EABC	4.64*E* − 16	6.34*E* − 16	1.06*E* − 16	EABC	2.50*E* + 02	2.93*E* + 02	2.32*E* + 01
JA-ABC5	2.67*E* − 16	5.30*E* − 16	8.66*E* − 17	JA-ABC5	2.08*E* + 02	2.85*E* + 02	1.26*E* + 01

*f*14	MIN	Average	STD DEV				

ABC	3.32*E* − 08	4.03*E* − 04	1.31*E* − 03				
BABC1	4.08*E* − 16	7.40*E* − 03	2.82*E* − 02				
IABC	2.72*E* − 16	1.06*E* − 15	2.41*E* − 15				
EABC	3.03*E* − 16	1.43*E* − 11	4.70*E* − 11				
JA-ABC5	2.57*E* − 16	3.70*E* − 03	2.03*E* − 02				

**Table 3:** Performance of optimization algorithms in solving RPO.

Algorithms	Ploss (MW)
SARGA	4.5740
PSO	4.6282
CLPSO	4.5615
EGA-DQLF	3.2008
ABC	1.5522
IABC	1.5185
BABC1	1.5215
EABC	1.5180
**JA-ABC5 **	**1.4985**
